# Domain General Sequence Operations Contribute to Pre-SMA Involvement in Visuo-spatial Processing

**DOI:** 10.3389/fnhum.2016.00009

**Published:** 2016-01-26

**Authors:** E. Charles Leek, Kenneth S. L Yuen, Stephen J. Johnston

**Affiliations:** ^1^Wolfson Centre for Clinical and Cognitive Neuroscience, School of Psychology, Bangor UniversityGwynedd, UK; ^2^Neuroimaging Centre, University Medical CentreMainz, Germany; ^3^Department of Psychology, Swansea UniversitySwansea, UK

**Keywords:** sequence processing, mental rotation, visuo-spatial transformation, supplementary motor area, domain general processing

## Abstract

This study used 3T MRI to elucidate the functional role of supplementary motor area (SMA) in relation to visuo-spatial processing. A localizer task contrasting sequential number subtraction and repetitive button pressing was used to functionally delineate non-motor sequence processing in pre-SMA, and activity in SMA-proper associated with motor sequencing. Patterns of BOLD responses in these regions were then contrasted to those from two tasks of visuo-spatial processing. In one task participants performed Mental Rotation (MR) in which recognition memory judgments were made to previously memorized 2D novel patterns across image-plane rotations. The other task involved abstract grid navigation (GN) in which observers computed a series of imagined location shifts in response to directional (arrow) cues around a mental grid. The results showed overlapping activation in pre-SMA for sequential subtraction and both visuo-spatial tasks. These results suggest that visuo-spatial processing is supported by non-motor sequence operations that involve pre-SMA. More broadly, these data further highlight the functional heterogeneity of pre-SMA, and show that its role extends to processes beyond the planning and online control of movement.

## Introduction

Visuo-spatial processing has most frequently been studied using variants of the classic Mental Rotation (MR) task in which observers make shape equivalence, or mirror-image, judgments about rotated images (e.g., Shepard and Metzler, 1971; Shepard and Cooper, 1982; Tarr and Pinker, 1990). In this task response latencies are often (but not always) found to increase as a function of stimulus angular disparity—an observation that has been taken as evidence for the existence of a spatial transformation mechanism in human vision (e.g., Shepard and Metzler, 1971; Shepard and Cooper, 1982; Tarr and Pinker, 1990; Leek, 1998a,b; Arguin and Leek, 2003; Leek and Johnston, 2006; Leek et al., 2007; Harris et al., 2008). The functional and neurobiological substrates of this mechanism remain unclear. One observation that has attracted considerable interest is the association between MR and the motor system (e.g., Wexler et al., 1998; Moreau, 2012, 2013; Moreau et al., 2012). For example, a number of functional imaging studies have shown involvement of premotor cortex—and most typically anterior supplementary motor area (pre-SMA) in visuo-spatial tasks (Kosslyn et al., 1994; Cohen et al., 1996; Tagaris et al., 1998; Sugio et al., 1999; Richter et al., 2000; Jordan et al., 2001; Lamm et al., 2001; Hanakawa et al., 2002; Vanrie et al., 2002; Johnston et al., 2004)—see Zacks (2008) for a review. Other evidence of motor system involvement has come from behavioral studies showing interactions between motor and MR (e.g., Wexler et al., 1998; Wohlschläger and Wohlschläger, 1998; Wohlschlager, 2001; Moreau, 2012, 2013; Moreau et al., 2012).

While the association between manual and MR has been described in terms of so-called “motor simulation” (e.g., Wexler et al., 1998), the precise functional contributions of motor areas to visuo-spatial processing tasks like MR have not been widely studied. Wexler et al. (1998) suggested that one component of this association is visuomotor anticipation—that is, functional processes involved in computing the visual consequences of movement also support visuo-spatial transformation operations. This is consistent with functional imaging evidence linking MR and pre-SMA—a region traditionally associated with the planning and online control of visually guided movement (Passingham, 1996; Picard and Strick, 1996; Tanji, 1996; Nachev et al., 2008, 2009). However, the SMA is neither an anatomically nor functionally homogenous area (e.g., Ashe and Ugurbil, 1994; Johansen-Berg et al., 2004; Lehéricy et al., 2004; Nachev et al., 2007, 2008, 2009; Leek and Johnston, 2009). The pre-SMA can be distinguished from the more caudal SMA-proper by distinct projections to the anterior and posterior striatum (e.g., Lehéricy et al., 2004). SMA-proper projects to dorsal premotor cortex (PMd), primary motor cortex (M1) and the spinal cord (Bates and Goldman-Rakic, 1993; Luppino et al., 1993; Lu et al., 1994; Tanji, 1996). In contrast, pre-SMA has cortical connections with the prefrontal cortex and receives ventral pathway input via the anterior infero-temporal cortex (Bates and Goldman-Rakic, 1993; Luppino et al., 1993; Lu et al., 1994). These areas, together with the supplementary eye fields (SEF) make up the supplementary motor complex or SMC (Luppino et al., 1993; Passingham, 1996; Picard and Strick, 1996, 2003; Tanji, 1996; Johansen-Berg et al., 2004; Nachev et al., 2007, 2008).

Of interest here is the potential link between other evidence highlighting the involvement of pre-SMA with sequence or serial chaining operations in motor and non-motor tasks, and visuo-spatial processing underlying MR. This evidence has shown that pre-SMA is active during sequence processing in both explicitly motor related tasks such as complex action planning (Tanji and Shima, 1994; Tanji, 1996; Nakamura et al., 1998; Kennerley et al., 2003) and motor response competition (Rushworth et al., 2002), as well as tasks without overt motor sequencing components such as serial number subtraction (e.g., Johansen-Berg et al., 2004) and sequence learning (Sakai et al., 1999). These observations motivate the hypothesis that pre-SMA activity during MR, or other visuo-spatial tasks, reflects the functional involvement of domain-general sequence processes in these tasks. This possibility is consistent with the idea that visuo-spatial processing routines, that underlie tasks like MR, rely on sequential operations related to vector transformation (e.g., the serial remapping of spatial locations across changes in stimulus orientation in MR tasks; Leek and Johnston, 2006, 2009).

The aim of the current study is to examine whether pre-SMA activity associated with visuo-spatial processing is linked to non-motor, domain general, sequence operations. We investigated this issue using fMRI in two studies involving separate groups of participants. Both studies used a localizer contrast to distinguish regions of pre-SMA associated with non-motor sequence processing (serial number subtraction) and those of SMA-proper that are associated with motor sequencing (repetitive button pressing). These tasks were based on previous work by Johansen-Berg et al. (2004). The two studies involved different tasks of visuo-spatial processing. Study 1 used a variant of a classic MR task in which observers were trained to recognize a sub-set of 2D novel shapes at a single canonical orientation. In a subsequent test phase they performed a recognition memory task in which, on each trial, a single object was classified as either a target or non-target at previously learned or unfamiliar orientations. Study 2 used a task of abstract mental grid navigation (GN; Hanakawa et al., 2002; Sawamoto et al., 2002). In this task observers viewed a 3 × 3 grid in which a start location was indicated by briefly outlining one grid square with a color cue. The grid was then removed from the screen and followed by a sequence of centrally presented arrow cues and placeholders. Observers were instructed to mentally compute movements of the cued square around the grid in response to presented arrow cues. The arrows indicated a movement of one square either left, right, up or down. At the end of the trial a response grid was shown indicating one of the nine possible locations end locations (that is, the grid location corresponding to movements from the start location given the arrow sequence). The task was to indicate whether the end location was correct or incorrect given the presented transformation sequence. Although the trial structure of this task is superficially very different from the MR task, the two tasks share a common reliance on the computation of visuo-spatial transformations. However, only the GN task, like serial subtraction, has an implicit sequential trial structure. This allowed us the possibility to examine patterns of overlap in pre-SMA activation during the MR task that can be attributed to sequence processing. Observers completing the GN task also undertook alternate blocks of a manual task involving repetitive button press responses to sequences of arrow cues—allowing us to identify regions of pre-SMA and SMA-proper associated with domain general sequence operations underlying the subtraction task, and sequence processes linked to repetitive motor activity.

## Materials and Methods

### Participants

In total 21 right-handed participants (7 male; Mean age = 26.7; *SD* = 5.2) were recruited from the local community subject panel. Participants were assigned randomly across the two studies (see below). All participants had normal or corrected-to-normal visual acuity and no prior history of developmental or acquired cognitive disability. The protocols for all experiments were approved by the Ethics Committees of the School of Psychology, Bangor University in accordance with the ethical standards laid down in the Declaration of Helsinki (1964). Informed consent was obtained from all participants prior to testing.

### Apparatus

The experiments were presented using a PC running E-Prime (Psychology Software Tools, PA, USA) software. Outside the scanner stimuli were shown on a 19″ TFT monitor running at a resolution of 1024 × 768 (60 Hz refresh), with responses collected via a standard USB keyboard. Inside the scanner stimuli were displayed via back-projection onto a screen at the head end of the scanner and were viewed through a mirror mounted on the head coil; responses were collected via a fiber-optic button box (Current Designs, Inc., PA, USA).

### Design and Procedure

In two studies participants performed a pre-SMA/SMA-proper sequence localizer task and one of two visuo-spatial transformation tasks. The studies are described below.

#### Study 1: Mental Rotation (MR)/Serial Subtraction vs. Button Press

Ten participants (3 male; mean age 26.1; *SD* = 5.6) completed Study 1. The participants completed two functional imaging runs of a localizer task and a separate MR task. The localizer tasks were based on those reported by Johansen-Berg et al. (2004) for delineating pre-SMA and SMA-proper, and used here to identify regions of pre-SMA associated with non-motor sequence processing. The task contrast involved interleaved trials of serial number subtraction and manual button pressing in response to identical trial sequences. There were a total of 18 trial presentations of serial subtraction/button press across the two functional imaging runs.

##### Serial subtraction

**Figure 1:**
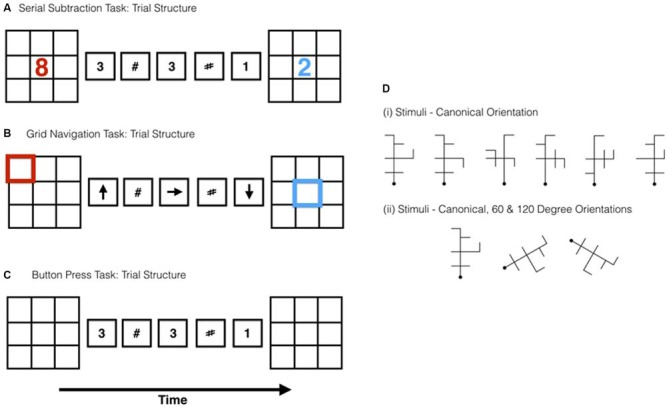
**Schematic representations of the trial structure for the (A) serial subtraction (B) grid navigation (GN) and (C) button press tasks. D(i)** Shows the stimuli used in the Mental Rotation (MR) task and **D(ii)** an example of the stimuli in each of the rotated positions.

Stimulus displays consisted of a black 3 × 3 grid drawn on a white background subtending 10° of visual angle. The grid served as the basis for the “start” and “response” displays in which a red integer was shown randomly in one of the nine square locations within each grid—see Figure 1A. Trial sequences also contained integers (Arial font) interleaved with hash (#) marks, both stimulus types subtended 4° of visual angle. Each trial started with a centrally presented task instruction screen (2500 ms) that informed the participant which of the two tasks within the given experiment (i.e., “button press” or “subtraction”) would be performed on the next trial. The trials were presented in an interleaved sequence with the first trial type counterbalanced across runs and participants. After the task instruction screen an empty grid was displayed (500 ms). The empty grid was followed by a “starting” grid that contained, in one randomly allocated square, a red number (3 s). The starting grid was followed by an ISI (500 ms) prior to the onset of the subtraction sequence. The subtraction sequence consisted of a total of five items and included a mixture of single numeric characters and hash marks. Each item in the subtraction sequence was shown centrally (1800 ms per item). The amount of number stimuli in the sequence varied between 1, 2 and 3, with the remaining items in the sequence being hash marks. The order of presentation of hash/number stimuli in the sequence was random. After the subtraction sequence ended a response grid was shown, with a target number presented, in blue, that was either “correct” or “incorrect” (50:50 split), as determined by the application of the preceding sequence on the starting number. The participant was asked to indicate via key press (counterbalanced across the subjects for handedness) whether the blue number in the response grid was the correct value arising from the serial subtraction of each number in the sequence from the starting number (e.g., Starting number = 10: 2 # # 4 *#* = 4). The participants were allowed 3 s to respond. Once the three second period had expired there was a randomized jittered delay (4, 6, or 12 s), during which a fixation cross was displayed, before the initiation of the next trial.

##### Button press

The button press task was visually identical except for the following modifications. There were no numbers presented in either the start or response grids. The task was simply to press down on both left and right response pad buttons when a number was presented in the subtraction sequence, and not to press when a hash mark was shown. Since there was no number presented in the response grid, there was no response made to the final grid. Figure 1C shows a schematic of the trial structure.

##### Mental rotation task

The experimental task used six novel antennae-like shapes based on those developed by Tarr and Pinker (1990)—see Figure 1D. Each stimulus comprised six line segments drawn with black lines on a white background, each subtending 5° of visual angle. For each stimulus a canonical “upright” orientation was defined in which the principal central axis, denoted at one end by a horizontal bar, was aligned with the vertical axis of the monitor. Non-canonical versions were created by rotating the principal axis ±60° and ±120° in the image plane (see Figure 1D). Prior to the start of the main experiment, outside of the scanner, the participants were asked to learn three of the six objects (counterbalanced across participants). Participants viewed the “to be learned” items for a period of 3 min along with the key that they would use to respond to them. The keys (1, 2 and 3) and hand position were selected so that the participant would associate each stimulus with a button press of the index, middle and ring fingers of their right hand. This was done so that they could more naturally transfer the learnt response outside the scanner to inside the scanner environment. Each item that was presented in the learning phase was shown in the canonical orientation only. Once the 3 min learning period was over, participants were tested to ensure that they had memorized the stimuli by undergoing five presentations of each object (15 trials total), presented in the canonical orientation only, and responded, via key press, which of the objects they believed it was (1, 2 or 3). Practice trials were initiated via pressing the spacebar which was then followed by a fixation (2 s) and an ISI (250 ms), before the presentation of the stimulus which remained onscreen for 1.5 s. Once the participant made their response a feedback screen was presented which informed them if they were correct or not. If they had answered incorrectly they were also told which stimulus it was. Participants had to achieve over 80% in this recognition test to proceed to the scanning phase of the experiment. This criterion was achieved by all participants. Additionally, participants were informed of the procedure for the localizer task (see above) and completed six practice trials of that task prior to entering the scanner. Inside the scanner, the participants performed two functional imaging runs of both the MR and the localizer tasks. The task run order was interleaved so that one block of the MR task preceded one block of the subtraction/button press localizer (see below).

In the scanner, the trial structure of the MR task was similar to the pre-scanning learning phase with the exception that there was no longer any feedback given at the end of each trial, trials were no longer self-initiated and they contained both previously learned and unfamiliar orientations of targets and distracters. A random inter-trial jitter of 2, 6, or 10 s was included to allow for the estimation of the BOLD response to each trial type. Participants responded using their right hand, and the numeric 1, 2 and 3 keys were replaced by three buttons that were in a linear arrangement on the MRI compatible button box. Before each task run participants were verbally instructed over the intercom which task was required.

#### Study 2: Grid Navigation/Serial Subtraction vs. Button Press

Eleven participants (4 male; mean age: 27.4 ± 5.2) completed Study 2. The participants completed two localizer tasks and an abstract mental GN task that, unlike MR, comprises an explicit requirement for both sequence processing and visuo-spatial transformation. There were 18 trial presentations of GN/subtraction/button press across the two functional imaging runs.

##### Serial subtraction and button press tasks

The subtraction task and button press task was the same in terms of trial structure and task requirements as the one used in Study 1.

##### Grid navigation task

The stimulus displays consisted of the same 3 × 3 grids used for the subtraction task—see Figure 1B. The trial structure and task was modified in the following ways: The starting grid was a 3 × 3 black grid where the outline border of one of the constituent squares was shown in red. The response grid was similarly constructed, but the border of the “target” square was highlighted in blue, instead of red. Additionally, a set of black arrows (4° visual angle) were created for the main GN sequence presentation, where the arrows could point in any of the four cardinal directions (up, down, left and right). The same hash marks used in the subtraction task were also used in the GN sequences. Each trial started with a centrally presented display (2500 ms) that informed the participant which task they would be performing (GN or button press). As before, trials were presented in an interleaved sequence with the first trial type counterbalanced across runs and participants. In this task participants had the same presentation sequence as in the subtraction task, with the following exceptions. Instead of a starting number, a start location was indicated in the starting grid by highlighting one of the nine squares in blue. The numbers in the following trial sequence were replaced by arrow cues (up, down, left, right) and hash marks (1800 ms per item). The final response grid was a black 3 × 3 grid with a single red highlighted tile. The task was to compute the transformation within the grid, prompted by the arrow cues, given the cued start location. No transformation was performed when the sequence item was a hash mark (e.g., Start location = top row left: →, # → # ← = Top row, middle).

On trials where the participant was asked to perform a button press, the procedure was identical to that in the subtraction vs. button press localizer task, i.e., no starting and response grid, with the exception that the participants pressed the button pad in response to the arrow stimuli, analogous to the requirement in the serial subtraction/button press task. Before each task run participants were verbally instructed over the intercom which task was required.

### Imaging Parameters

MRI scanning was performed on a Philips Achieva 3T MRI scanner in the School of Psychology, Bangor University. A SENSE head coil was used with a SENSE acceleration factor of 2. For the localizer and GN tasks 232 functional images (2.5 mm^2^ in-plane resolution, TR = 2 s, TE = 35 ms, 90° flip angle, 3 mm slice thickness, 28 slices) were collected. The first four images were discarded to allow for T1 saturation effects, giving a total of 228 functional images collected for each of the experimental runs for subsequent analysis. Additionally a high resolution (1 mm isotropic voxels) T1 weighted image was collected.

### Data Analysis

#### Data Pre-Processing

Data were analyzed using BrainVoyager QX 2.2 software (Brain Innovation, B.V., Maastricht, Netherlands). The functional imaging data were pre-processed using the following steps. Motion correction was applied (sinc interpolation) to compensate for subject head movement during the scans, normalized to the Talairach stereotaxic space (Talairach and Tournoux, 1988) and a 4 mm 3D Gaussian smoothing kernel applied. Additionally, both high (GLM-Fourier method: 2 sines/cosines) and low (Gaussian filter: 3 s FWHM) pass filters were applied to remove unwanted signal noise from the data. The T1 weighted anatomical image was also normalized to Talairach space.

#### Whole Brain Analysis

The functional imaging data were submitted to a random effects general linear model (GLM). For the SMA localizer task regressors were constructed for the following events, “instruction”, “button press”, “subtraction” and “response”. The GN experimental task (Study 2) used the same regressors as the localizer task, with the exception that there was no “subtraction” regressor, but a “GN” regressor in its place. The instruction regressor covered the first 3 s of the trial, button press/GN covered the 12 s of the trial constituting the trial sequence, and the response regressor covered a 4.5 s period with the onset at the initiation of the response screen. For the MR experimental task (Study 1), regressors were constructed for each stimulus orientation: 0° (canonical), 60° and 120° (non-canonical), with the onset corresponding to image presentation (duration = 1.5 s). Events were not created for the individual stimuli, but based only on the presented orientation of the stimuli. All regressors were convolved with a hemodynamic response function that models the expected delay and generic shape of the blood oxygen level dependent (BOLD) signal. Additionally, for all tasks, to account for any residual movement related signal changes, the motion correction parameters (three translational, three rotational) were also included as regressors of no interest. The resultant statistical maps output from the GLM were thresholded at a value of *p* < 0.05 and corrected for multiple comparisons using cluster-level threshold estimation. This form of multiple comparison correction involves determining the maximum cluster size that we might expect to result by chance alone, given the number of statistically significant voxels accepted at the individual voxel-level threshold of *p* < 0.05 uncorrected. For the cluster threshold correction we also used a *p* < 0.05 acceptance threshold, i.e., the probability that the given cluster size could occur by chance is less than 5%.

#### Region of Interest (ROI Analyses)

All regions selected for subsequent ROI analysis were created such that all supra-threshold voxels within a 10 mm^3^ region, centered on the peak voxel, were included. The ROI analyses involved extracting the beta values resulting from the GLM analysis for each of the conditions under test and contrasting the values using paired samples *t*−tests.

## Results

### Behavioral Data

**Figure 2:**
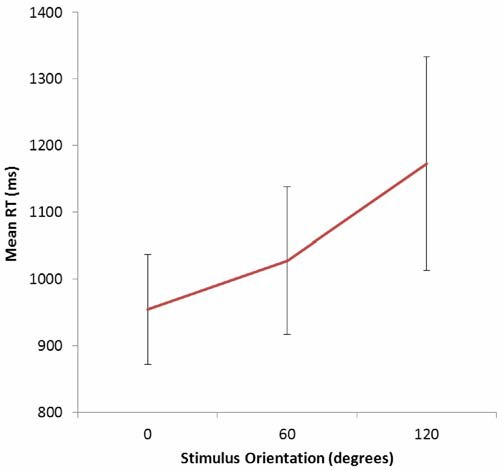
**Mean RTs as a function of stimulus orientation in the mental rotation task.** Bars show standard error of the mean.

For the MR task Overall accuracy was 89.63%, SE = 2.77%. Mean RTs for correct responses increased with angular disparity—see Figure 2; *F*_(2,10)_ = 4.98; slope = 1.81 ms/deg; *p* = 0.03 (two-tailed) consistent with previous reports of MR effects for this class of stimulus (e.g., Tarr and Pinker, 1990; Johnston et al., 2004). Accuracy data showed the same pattern as RTs: Mean correct (SE): 0° = 28/30 (0.79); 60° = 26.83/30 (0.93); 120° = 25.83/30 (0.77), although there was no significant difference across orientations.

In the GN task overall accuracy was 73.7% (SE = 6.8%) indicating that, like above, the participants were engaging with the task yet the task itself was non-trivial. In the subtraction localizer task overall accuracy was 93.9% (SE = 3.08%).

### fMRI Analysis: SMA Localizers—Subtraction vs. Button Press

Table 1 shows the areas of significant activation for the contrast of subtraction vs. button press tasks.

**Table 1:** **Clusters of activity for Contrast 1: Subtraction—Button Press task, *p* < 0.05 (cluster correction *p* < 0.05)**.

Area	Hemisphere	Talairach co-ordinate			Voxel *t*−value
		*x*	*y*	*z*	
Thalamus	L	−23	−29	−1	4.6
Middle frontal gyrus/pre-central gyrus (BA 6/4)	R	17	−5	23	2.9
**Medial frontal gyrus (BA 6)**	**L**	**−8**	**9**	**48**	**5.6**
Thalamus	R	20	−28	−2	3.9
Middle frontal gyrus (BA 8/9)	R	26	42	31	2.8
Thalamus	R	18	−28	3	4.0
Inferior frontal gyrus (BA 45)	R	30	17	9	5.5
Middle occipital gyrus (BA 19)	R	31	−78	11	4.6
Superior/inferior parietal lobe (BA 7/39)	L	−26	−67	32	5.6
Superior/inferior parietal lobe (BA 7/39)	R	31	−66	33	6.6
Middle frontal gyrus (BA 6)	R	38	−3	33	4.0
Middle temporal gyrus (BA 21)	R	48	−32	−1	3.6
Lingual gyrus (BA 18)	R	10	−79	−8	5.1
Lingual gyrus (BA 18)	L	−13	−70	−1	5.6
Caudate	L	−12	9	8	4.9
Caudate	R	13	10	5	5.0
Inferior frontal gyrus (BA 45/47)	L	−27	18	10	4.6
Superior/inferior parietal lobe (BA 7/40)	L	−40	−55	38	8.1
Inferior frontal gyrus (BA 9)	R	41	24	33	5.8
Middle frontal gyrus (BA 6)	L	−41	−4	41	3.7

*Pre-SMA activity highlighted in bold*.

These correspond to regions that show greater activity when performing serial subtraction relative to the button press task. The results correspond well with those reported by Johansen-Berg et al. (2004). Of particular relevance here is the cluster of activation in the left medial frontal gyrus (Talairach co-ordinate: −8, 9, 48), which is consistent with the connectivity-defined region for pre-SMA which extends along the dorso-medial frontal gyrus rostrally from the vertical anterior commissure (VCA) between 0–15 mm (Johansen-Berg et al., 2004, Figures 1, 4). Additionally this contrast revealed significant activations in bilateral superior and inferior parietal lobes, and in the prefrontal cortices, specifically bilateral middle and inferior frontal gyri.

**Figure 3:**
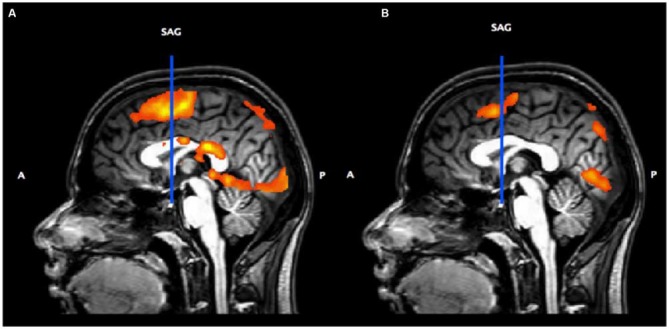
**(A)** Sagittal slice (midline) showing the vertical anterior commissure (VCA) line and overall activation for the Subtraction and Button Press tasks (*p* < 0.05, *p* < 0.05 cluster correction threshold) from the Subtraction localizer task. **(B)** Contrast of Subtraction vs. Button Press.

Figure 3A shows the activation associated with the Button Press and Subtraction Tasks (vs. implicit baseline) overlaid on a sagittal section. This shows that our tasks activate medial premotor cortex including pre-SMA and SMA proper. Figure 3B shows the result of the contrast of Subtraction vs. Button Press. The vertical blue line represents a plane passing through the anterior commissure (VCA); medial pre-motor activity anterior to the VCA line is considered pre-SMA and is where the locus of peak activity for the Subtraction vs. Button Press task is found.

### fMRI Analysis: Mental Rotation Task

The aim of this analysis was to determine the extent to which activity associated with visuo-spatial transformation in this task overlaps with pre-SMA activity associated with the sequential subtraction task as defined by the subtraction vs. button press contrast (see above). The contrast of interest was therefore between canonical vs. non-canonical target presentations (where it is assumed that visuo-spatial transformation must be used to map non-canonical views onto canonical views during target identification). The significantly active areas revealed by this contrast are shown in Table 2.

**Table 2:** **Clusters of activity for Contrast 2: MR task (Transform—Canonical), *p* < 0.05 (cluster correction *p* < 0.05)**.

Area	Hemisphere	Talairach co-ordinate			Voxel *t*−value
		*x*	*y*	*z*	
Pre-central gyrus (BA 6)	L	−31	6	24	4.8
Middle frontal gyrus (BA 8)	L	−28	24	30	4.9
Pre-central gyrus (BA 6)	L	−34	4	19	4.0
Superior frontal gyrus (BA 6)	L	−21	−7	54	3.9
Superior/inferior parietal lobe (BA 7/40)	L	−20	−62	28	5.5
Fusiform gyrus (BA 37)	L	−28	−39	2	3.4
**Medial frontal gyrus (BA 6)**	**L**	**−2**	**6**	**56**	**3.3**
Superior/inferior parietal lobe (BA 7/39)	R	20	−66	29	4.0
Superior/inferior parietal lobe (BA 7/39)	R	25	−64	17	4.5
Middle frontal gyrus (BA 9/46)	R	29	39	31	4.9
Inferior frontal gyrus/insula (BA 44)	R	33	12	14	4.1
Superior/inferior parietal lobe (BA 7/40)	R	44	−47	50	2.6
Superior/inferior parietal lobe (BA 7/40)	L	−33	−55	46	4.5
Fusiform gyrus (BA 18)	L	−33	−75	−7	3.1
Inferior temporal gyrus (BA 40)	L	−30	−44	26	5.6

*Pre-SMA activity highlighted in bold*.

The results confirm a significant cluster of activation in the pre-SMA (Talairach co-ordinate: −2, 6, 56) that corresponds closely to the pre-SMA ROI delineated by the localizer contrast. This confirms pre-SMA involvement in MR, and shows that the areas of activity within this region associated with sequential processing are active during the MR task. To clarify that the region of pre-SMA active in the MR task was the same as that found in the subtraction vs. button press contrast, a ROI analysis was performed. The ROI constituted a 10^3^ voxel region with the ROI centered on the voxel that showed the highest level of significance in the MR task contrast: canonical vs. non-canonical. A paired *t*-test showed that this same cluster was significant in the subtraction vs. button press task (*t*_(9)_ = 3.27, *p* < 0.01).

**Figure 4:**
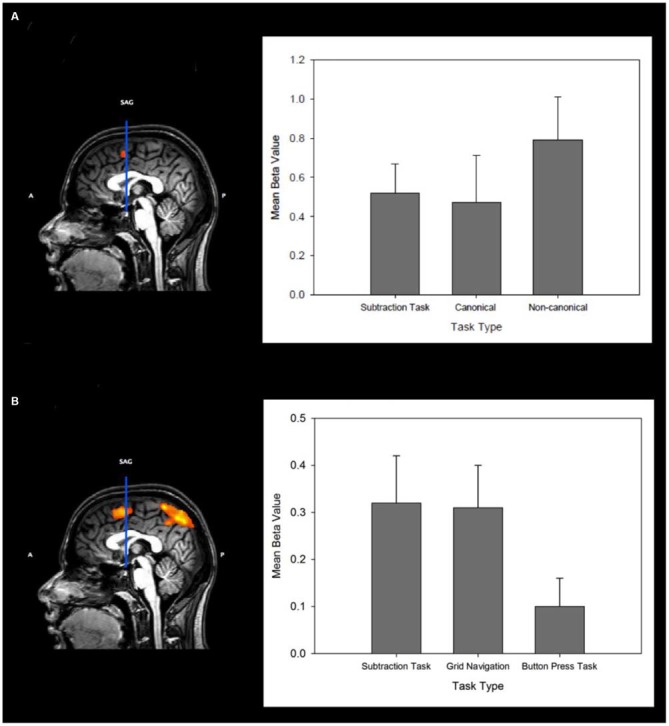
**(A)** Left: Sagittal slice (midline) showing the VCA line and significant clusters of activation (*p* < 0.05, *p* < 0.05 cluster correction threshold) for the Mental Rotation Task (MR). Right: Mean beta values from the pre-SMA region for the subtraction task, and for the canonical and non-canonical MR conditions. Error bars indicate the standard error of the mean. **(B)** Left: Sagittal slice (midline) showing the VCA line and significant clusters of activation (*p* < 0.05, *p* < 0.05 cluster correction threshold) for the GN task. Right: The mean beta values from the pre-SMA region for the subtraction task, Grid Navigation task and Button Press task. Error bars indicate the standard error of the mean.

Figure 4A (left) shows in sagittal section the cluster for the MR task within the pre-SMA. Figure 4B (right) shows the mean beta values for the subtraction and 0° (canonical) vs. 60°/120° (non-canonical) conditions for the pre-SMA defined in the ROI analyses. As shown in Table 2 the analysis also showed a network of other regions typically found in studies of MR. These include the superior and inferior parietal cortex (bilaterally), prefrontal cortex and the dorso-lateral prefrontal cortex. Notable also is that this variant of the MR task, which requires the prior memorization and subsequent retrieval of a stored shape representation, also elicited greater activity during recognition from non-canonical views of regions in the ventral occipital-temporal cortex.

### Additional Analyses

Two additional analyses were conducted to: (i) investigate the possible contribution of eye movement patterns to the observed pre-SMA activity in MR and (ii) whether modulation of the pre-SMA sequence processing voxel cluster during MR could be accounted for by variation in behavioral response latencies.

#### Eye Movements

In order to examine whether eye movements could account for the BOLD-related responses found in pre-SMA, we conducted an off-scanner study (*N* = 12 participants, 5 male, 7 female, mean age 28.8 ± 4.9 years) to measure eye movement patterns using the same MR task, stimulus, design and trial procedure used in the scanner. Analyses of eye movement data comparing mean saccade frequency and stimulus orientation showed no effect of stimulus orientation, (0°*M* = 2.13, *SD* = 0.14; 60°*M* = 2.16, *SD* = 0.14; 120°*M* = 1.97, *SD* = 0.1); *F*_(2,18)_ = 0.45, ns, and no correlation between stimulus orientation and mean saccade frequency, $r_{\text{ adj}}^{2}$ = −0.03, *F*_(1,28)_ = < 1, ns. This suggests that any differences in saccade frequency cannot account for the association between MR and pre-SMA activity.

#### Pre-SMA Sensitivity to Response Latencies

We additionally examined whether the modulation of the pre-SMA sequence processing voxel cluster found in the MR task could be accounted for solely by RTs (i.e., “time-on-task”) because mean RTs in the non-canonical condition were longer than in the canonical condition. If this were the case we would expect to find a correlation between mean Beta values and RTs in the *non-canonical* condition alone. There was no evidence for this; *r* = −0.072, (*p* < 0.842). Mean Beta = 0.767 (*SD* = 0.64), Mean RT = 1297 ms (*SD* = 259). Neither was there any correlation between mean Beta and overall observer RTs (collapsed across canonical and non-canonical conditions in the MR task): *r* = −0.030, (*p* < 0.935). Mean Beta = 0.667 (*SD* = 0.65), Mean RT = 1229 ms (*SD* = 205). In contrast, the same analyses for an SMA ROI based on the button pressing task (Mean coordinates; *X* = −0.3 (SE = 1.1); *Y* = −20.3 (SE = 2.4), *Z* = 50.7 (SE = 1.7) showed a strong correlation between mean beta and RT; *r* = 0.60, *p* < 0.03. Clearly, pre-SMA and SMA are not showing the same pattern of responses in the task contrasts.

### fMRI Analysis Grid Navigation vs. Button Press

The aim of this analysis was to determine whether the pre-SMA activity associated with sequential subtraction and MR would also be found in the GN task. This was examined by a contrast of the activation during GN with that for button pressing. The results are shown in Table 3.

**Table 3:** **Clusters of activity for Contrast 3: Grid Navigation task—Button Press task, *p* < 0.05 (cluster correction *p* < 0.05)**.

Area	Hemisphere	Talairach co-ordinate			Voxel *t*−Value
		*x*	*y*	*z*	
Middle temporal gyrus/inferior temporal gyrus (BA21/37)	L	−55	−45	0	3.3
Middle frontal gyrus (BA 8/9)	L	−38	21	35	4.0
Inferior frontal gyrus (BA 45)	L	−29	21	10	3.3
Inferior frontal gyrus/insula (BA 45)	R	29	17	10	4.0
**Medial frontal gyrus (BA 6)**	**L**	**−2**	**8**	**56**	**5.1**
Middle temporal gyrus (BA 21)	R	46	−37	3	3.3
Superior parietal lobe (BA 7)	L	−19	−77	40	11.8
Superior parietal lobe (BA 7)	R	15	−70	39	15.5
Middle frontal gyrus (BA 6)	R	41	−5	40	6.1
Middle frontal gyrus (BA 6)	L	−43	2	33	6.6
Inferior temporal gyrus (BA 37)	L	−48	−52	−8	3.2
Inferior temporal gyrus (BA 37)	R	50	−54	−5	4.2
Middle occipital gyrus (BA 19)	L	−31	−79	15	5.5
Middle occipital gyrus (BA 19)	R	30	−73	15	4.9
Superior temporal gyrus (BA 22)	L	−52	−47	15	3.4
Inferior frontal gyrus (BA 44)	R	52	7	13	3.0

*Pre-SMA activity highlighted in bold*.

The analysis confirms a significant cluster of activation in the pre-SMA (Talairach co-ordinate: −2, 8, 56) the center of which is virtually identical to that found for the MR task. This shows that the focus of cortical activation in pre-SMA associated with MR is also found in GN despite very different task procedures. In an additional analysis, analogous to that performed above for the MR task, we used the cluster of activity in pre-SMA revealed by the contrast of GN vs. button press as a ROI for the subtraction vs. button press contrast to confirm that the same area was being utilized in both tasks. A paired *t*-test, using the beta values extracted from this region for each of our predictor variables, confirmed that the same cluster of activity was significantly more active for the subtraction task compared with button press (*t*_(10)_ = 3.24, *p* < 0.01). That is, there was significantly more activity during the transformation periods of GN (mean beta = 0.31 ± 0.09), and subtraction (mean beta= 0.42 ± 0.13) tasks than during the motor transformation periods of button press (mean beta = 0.1 ± 0.06) in pre-SMA. Figure 4B (left) shows in sagittal section the significant cluster for GN within the pre-SMA. Figure 4B (right) also shows the mean beta values for the subtraction, GN and button press tasks within this ROI.

Inspection of Table 3 also shows a range of other areas associated with performance of the GN task. As with MR, these include bilateral superior and inferior parietal regions, as well as bilateral inferior frontal and the left anterior middle frontal gyrus. Contrary to the subtraction task there are also several clusters of activation seen in bilateral middle occipital cortex, bilateral inferior and middle temporal cortex, and superior and inferior temporal regions but not the (left) fusiform gyrus.

## Discussion

The goal of this study was to examine the hypothesis that pre-SMA activity during visuo-spatial tasks derives from its involvement in domain general sequence processing operations. Sequence processing areas of pre-SMA were localized using a serial numerical subtraction task vs button press contrast previously described by Johansen-Berg et al. (2004). As in that study we found predominant activity in pre-SMA associated with serial subtraction anterior to the VCA (*y* = 0) line, while the button press task elicited activity predominantly in SMA-proper. We then extracted an ROI based on pre-SMA activity during serial subtraction and analyzed whether the BOLD response in this region is also modulated during a MR task in which observers made recognition memory judgments about novel 2D objects shown at either familiar or unfamiliar orientations. The results showed that activity within this region of pre-SMA associated with sequence processing was modulated by visuo-spatial transformation during MR. Further evidence was provided by data from the GN task. Although the trial structure of this task was very different from MR, the two tasks share a common requirement to compute visuo-spatial transformations. However, only the GN task, like serial subtraction, has an implicit sequential trial structure. This allowed us to examine patterns of overlap in pre-SMA activation during MR that can be attributed to sequence processing. The results showed very similar patterns of activation within the pre-SMA cluster associated with serial subtraction in MR and GN.

These findings provide new evidence about the functional processes that support MR, and further elucidate the computational mechanisms underlying motor system involvement in visuo-spatial tasks (e.g., Wexler et al., 1998; Wohlschläger and Wohlschläger, 1998; Wohlschlager, 2001; Wraga et al., 2003, 2010; Moreau, 2012, 2013; Moreau et al., 2012). They are also relevant to current debates about the involvement of traditional motor areas in non-motor functions (e.g., Nachev et al., 2008, 2009; Passingham et al., 2010). We consider each of these issues in turn.

First, our results suggest that the spatial transformation operations required for determining shape identity across image rotations recruit sequential processing routines. One hypothesis, outlined by Leek and Johnston (2009), is that the pre-SMA is part of a larger cortical network including DLPFC and the parietal cortex that supports visuo-spatial processing through the computation of vector transformations that allow spatial mappings between corresponding feature coordinates. In MR (whether this involves determining shape equivalence between two rotated images, or matching a rotated image to a stored shape representation) this account maintains that image alignment is realized by the transformation of feature coordinates within a spatial coordinate system. At a neurophysiological level, these operations may be based on a neuronal population vector (Georgopoulos et al., 1983; Pellizzer and Georgopoulos, 1993; Georgopoulos and Pellizzer, 1995; Pellizzer, 1996). Within the context of this hypothesis, the current results suggest that spatial transformation processes are sequential in operation and dependent on vector length. This, in turn, may reflect fundamental limitations of working memory capacity. This suggestion is supported by some recent evidence from Hyun and Luck (2007) who found the speed of spatial transformation during MR was impaired during the simultaneous performance of a visual working memory task requiring the maintenance of object features over an intervening delay. They speculated that visual working memory contributes to MR by providing temporary storage during spatial transformation.

Second, the current findings linking sequential processing in pre-SMA and MR help to elucidate further the functional connection between the motor system and visuo-spatial processing. Previous research has shown that the simultaneous performance of manual and MR tasks—under some conditions, can produce interference effects consistent with motor system involvement in visuo-spatial processing (e.g., Wexler et al., 1998; Wohlschläger and Wohlschläger, 1998; Wohlschlager, 2001; Wraga et al., 2003, 2010; Moreau et al., 2012; Moreau, 2012, 2013). Our results suggest that this functional link may, at least in part, be based on the shared use of sequence processing routines to which pre-SMA contributes.

Third, more broadly, the present results are also relevant to current debates about functional specialization and motor processing within the medial premotor cortex (e.g., Nachev et al., 2008, 2009; Passingham et al., 2010). We have presented evidence that the pre-SMA contributes to visuo-spatial transformation through the recruitment of sequencing processes. Thus, these processes appear to contribute to both motor and non-motor related activity—supporting the hypothesis that premotor function is not solely related to motor behavior. While sequence processes may support motor tasks when required, the same operations may also be recruited for a broader range of non-motor, functions including visuo-spatial transformation. These non-motor functions may contribute to a range of activities including navigation, object tracking, attention shifts as well as perception and object recognition (Leek and Johnston, 2009). This characterization proposes a relatively abstract functional role for pre-SMA—at least in relation to sequence processing routines. In addition, it defines, and predicts, a domain of functional involvement of pre-SMA in a diverse range of cognitive tasks requiring visuo-spatial transformation operations, as well as deficits in the performance of such tasks associated with pre-SMA dysfunction. Some support for the latter prediction comes from recent studies of Parkinson’s disease (PD)—a degenerative disorder caused by dopamine depletion in the basal ganglia, and associated dysfunction of pyramidal neurons in the pre-SMA (MacDonald and Halliday, 2002). Consistent with a putative role for pre-SMA in abstract visuo-spatial processing, PD patients have been shown to demonstrate deficits in spatial transformation (e.g., Lee et al., 1998; Kerai et al., 2012; Sawamoto et al., 2002)—although these deficits, as might be expected, are not always associated with sequencing processing impairments (e.g., Leek et al., 2014).

Pre-SMA is also associated with other functions that may be relevant to understanding its putative contribution to visuo-spatial processing. In particular, other studies have shown pre-SMA involvement in inhibitory control processes that underlie, for example, our ability to refrain from particular responses (e.g., Rushworth et al., 2007; Chen et al., 2009; Juan and Muggleton, 2012; Yu et al., 2015). Inhibitory control processes may also contribute to complex visuo-spatial tasks that involve determining a sequential transformation operation required to compute mappings between feature locations (as in MR) or shifts of spatial location in response to external cues (as in the GN task). We cannot determine the precise contribution of such inhibitory processes from the current data, but this possibility merits future investigation.

Finally, we consider one alternative account of the results, namely that the overlapping pre-SMA activations associated with serial subtraction, MR and GN may be determined solely by saccadic eye movements. There are several lines of evidence against such an account. First, the locus of pre-SMA delineated by the subtraction vs. button press contrast lies well outside of the range of coordinates for the SEF in humans (e.g., Grosbras et al., 1999) and of course, the frontal eye fields [Paus, 1996: *x* (−24 to −40), *y* (−6 to 1), *z* (44 to 48)]. Grosbras et al. (1999) have precisely delineated human SEF anatomically as lying on the upper part of the paracentral sulcus and plotted the variability across subjects (see Grosbras et al., 1999—Table 2) as: *x* (−4 to −12), *y* (−2 to −24), *z* (48 to 52). Note that despite greater than 2 cm variation along the rostral-caudal axis the most anterior coordinate for SEF lies posterior to the VCA line, within SMA-proper. Additionally, during the subtraction task observers centrally fixated throughout the period of task dependent BOLD acquisition. Second, although completed off-line, we showed that there were no differences in saccade frequency during the MR task when observers were presented with upright or rotated stimuli.

In conclusion, we examined the functional contribution of domain general, non-motor, sequence operations of the pre-SMA to visuo-spatial processing. Non-motor sequence operations in pre-SMA, and activity in SMA-proper associated with motor sequencing, were functionally localized by contrasting activation in response to serial number subtraction and sequential button pressing. BOLD responses in these regions from two tasks of visuo-spatial transformation were also measured. The results showed overlapping activation in pre-SMA for serial subtraction and both visuo-spatial tasks. These results suggest that visuo-spatial processing is supported by domain general, non-motor, sequence operations in pre-SMA. More broadly, the data highlight the functional heterogeneity of pre-SMA, and show that its role extends to domain general processes beyond the planning and online control of movement.

## Author Contributions

All authors were involved in study design, data analysis and manuscript preparation. We have read and approved the manuscript and agree to be accountable for all aspects of the work in ensuring that questions related to the accuracy or integrity of any part of the work are appropriately investigated and resolved.

## Conflict of Interest Statement

The authors declare that the research was conducted in the absence of any commercial or financial relationships that could be construed as a potential conflict of interest.
